# Biodegradation of PAHs by *Burkholderia* sp. VITRSB1 Isolated from Marine Sediments

**DOI:** 10.1155/2015/867586

**Published:** 2015-10-28

**Authors:** T. Revathy, M. A. Jayasri, K. Suthindhiran

**Affiliations:** Marine Biotechnology and Bioproducts Laboratory, School of Biosciences and Technology, VIT University, Vellore, Tamil Nadu 632014, India

## Abstract

The polycyclic aromatic hydrocarbons (PAHs) pollution to the environment is a major threat to the living organisms, and hence the degradation of these PAHs is necessary. Studies on PAHs degrading bacteria have focussed on terrestrial microbes and the potential of marine derived microbes is undermined. Herein we report the isolation and characterization of PAHs degrading *Burkholderia* sp. from lagoon sediments collected at the Southern coast of India. The strain was Gram negative, rod-shaped, motile, and ∼2–5 *μ*m in length. Based on the phylogenetic data the strain was identified as *Burkholderia* and designated as VITRSB1. Initial PAHs degradation ability of the strain was assessed using basal salt medium supplemented with diesel, kerosene, toluene, aniline, naphthalene, and phenol. The strain was found to be effectively degrading kerosene, diesel, toluene, and aniline even at higher concentration (1%). However, naphthalene and aniline were degraded only at lower concentration (0.1%) and phenol, camphor, and DAP inhibited the growth of the strain. Furthermore, the degraded end products of the PAHs were determined using FTIR. Notably, none of the end products were found to be toxic to the biosphere. Our results indicate that the isolated *Burkholderia* sp. could be a prospective candidate for the effective degradation of selective PAHs.

## 1. Introduction

Polycyclic aromatic hydrocarbons (PAHs) are produced as a result of both natural and human activities and are considered as environmental contaminants due to their toxic nature [1]. PAHs are formed as a result of incomplete combustion of organic materials such as fossil fuels. The sources of PAHs include motor vehicle exhausts, industrial activities, and coal burning [2]. The occurrence of PAHs in ambient air is an increasing concern because of their carcinogenicity and mutagenicity [3]. Although emissions and allowable concentrations of PAHs in the air are now regulated, the health risk posed by PAH exposure suggests a continuing need for their control through air quality management [3]. PAHs are possible contaminants in some former industrial sites, representing a potential risk to human health if these sites are converted to residential areas.

The bioremediation process is the accepted process for the reinstatement of PAH's contaminated groundwater and sediments [4, 5]. Biodegradation of petroleum hydrocarbons in oil and other nonaqueous phase liquids by bacterial cultures has been reported in both batch and field scale studies [6, 7]. Many bacterial species are known to degrade PAH [1, 8, 9]. Even though bioremediation is economic; few bacteria are reported to efficiently degrade high molecular weight PAHs. So there is an urgent need to find out novel strains for the degradation of high molecular weight PAHs. Among various bacteria,* Burkholderia* sp. is known to degrade a variety of environmental contaminants [1].* Burkholderia *are found both in soil and in water [10, 11].* Burkholderia cepacia* complex is a group of nine closely related bacterial species that have useful properties in the natural environment as plant pest antagonists, plant growth promoters, and degradative agents [12, 13]. Many species of* Burkholderia*, namely,* B. cepacia*,* B. vietnamiensis*, and* B. ambifaria*, have been reported to have biodegradation potential [13, 14]. In recent years,* B. cepacia *has been studied extensively and reported as a potential hydrocarbon degrading bacteria. It has been demonstrated as an excellent n-alkane degradation characteristics [7].* Burkholderia* sp. FDS-1 has been isolated and characterized for the degradation of fenitrothion, a nitro phenolic pesticide [15]. Additionally,* Burkholderia* have also been used as a plant growth promoting rhizobacteria because several mechanisms related to plant growth promotion were detected in this genus [16]. Nevertheless, isolation and exploitation of* Burkholderia* from marine ecosystem are scanty. In this study, a* Burkholderia* strain was isolated from Chavara lagoon located at the Southern coast of India. Considering the organism's potential for biodegradation, it was evaluated for the biodegradation of PAHs: diesel, kerosene, naphthalene, toluene, phenol, aniline, camphor, and a fertilizer, DAP complex.

## 2. Materials and Methods

### 2.1. Chemicals and Media

The PAHs used for the study were naphthalene, toluene, diesel, aniline, naphthalene, camphor, kerosene, and DAP complex (local purchase). The stock solutions of the PAHs were prepared as reported by [17]. The basal salt media (BSM) (Himedia, India) were used for the isolation and cultivation of* Burkholderia* sp. [18, 19] basal salt medium.

### 2.2. Sample Collection and Enrichment

Sediment samples were collected from the Ashtamudi lagoon, Chavara, located at the Arabian Coast of India (lat.: 8°57′48′N; long.: 76°33′43′E). Sediment samples collected from a depth of 5 cm were transferred into sterile plastic bottles and transported to the laboratory aseptically. The basal salt media components were added to the sediment sample bottles containing 100 mL of sediment water and incubated in dark for one week at 28°C. Then the sediment samples were serially diluted and plated on BSM media containing different PAHs (0.1% to 1%). The agar plates were incubated at 30°C for 2 days. The isolate that has grown on at least 3 PAHs containing media was chosen for further studies. The resistant isolate was subcultured repeatedly in BSM media to obtain the pure culture. The inoculum was prepared by culturing the isolate in BSM media at 30°C for 2 days. After incubation, the culture was centrifuged at (10621 ×g) for 10 min. The harvested cells were washed and reconstituted with BSM media and used as inoculums for further studies.

### 2.3. Taxonomy

The phenotypic characters of the strain were carried out as described earlier [20]. The fully grown culture was taken and centrifuged at 8000 ×g for 10 min. The obtained pellet was washed with potassium phosphate buffer (pH 7) for 3 times. Further, the pellet was fixed with 5% formaldehyde for 1 hour and washed again with phosphate buffer (pH 7). The pellet was lyophilised using freeze dryer SKL-12N (Lark, Chennai, India). The samples for TEM were prepared by directly applying the lyophilised cell pellets to copper grids and allowed to dry for 2 hr. These dried copper grids were then directly observed under TEM (TECHNAI10-Philiphs). The DNA of the strain was isolated using Hipura bacterial DNA isolation and purification kit (Himedia, India). The 16S rRNA gene was amplified using a Veriti 96-Well Thermal Cycler (Applied Biosystem Foster City, CA). PCR amplification was performed with primer set FC27 (5′-AGAGTTTGATCCTGGCTCAG-3′) and RC1492 (5′-TACGGCTACCTTACGACTT-3′) (Sigma Aldrich, USA). The methodology for sequencing was adapted from the earlier reports [21]. BLAST search was performed using NCBI database and multiple sequence alignment was carried out in ClustalX [22]. The phylogenetic tree was generated using the neighbour joining method [23] using MEGA 6 software [24]. Bootstrap values were calculated with 1000 replicates.

### 2.4. Primary Screening

A loopful of culture was inoculated into the BSM media containing different PAHs such as diesel, kerosene, toluene, aniline, naphthalene, phenol, camphor, and DAP complex. All the test compounds were syringe filtered. A stock solution of naphthalene was prepared in methanol (200 *μ*g/mL) and camphor was prepared in ethyl acetate (0.2 g/mL). DAP complex stock solution was prepared in water (0.5 g/mL). 1 mL and 0.1 mL of diesel, kerosene, toluene, aniline, and phenol and 1 mL of stock solution of naphthalene, camphor, and DAP complex were supplemented in BSM agar. The plates were incubated at 30°C for 48 hours.

### 2.5. Degradation Study in Liquid Culture

The ability of the strain to degrade the PAH is evaluated by inoculating it in BSM broth containing different PAHs (0.1% and 1%). About 1 mL of the compound (1%) was added to 20 mL BSM broth. To this, 100 *μ*L of primary inoculum was added and incubated for 48 hours at 30°C and kept in shaker and observed for growth. The culture was centrifuged at (6797 ×g) for 10 min and the supernatant was lyophilized (Lark Penguin Freeze Dryer, India). The lyophilized samples were analysed using FTIR (Schimadzu, Japan).

## 3. Results and Discussion

PAHs are group of compounds composed of fused aromatic rings that are highly stable and carcinogenic [25]. They are formed by the pyrolysis and pyrosynthesis of organic molecules. All the PAHs are known to cause cancer on prolonged exposure [26, 27]. Various methods have been employed to remove the PAHs including volatilization, chemical oxidation, and bioaccumulation [28]. Bacteria are one of the hydrocarbon degrading agents in the environment and their degradation potential depends upon the metabolic capability [29]. The potential approach for the remediation of PAHs is using the indigenous bacteria from the contaminated site. Though there have been several studies on PAH degrading bacteria from terrestrial ecosystem, studies on bacterial isolation from marine sites are very few. This study focussed on the screening and isolation of PAH degrading bacteria from Ashtamudi lagoon, Chavara. Chavara is situated on the Arabian Coast, Kerala, (lat.: 8°57′48′N; long.: 76°33′43′E). Chavara contains several lakes and lagoons. The seashore of Chavara is rich in titanium [30]. Kerala Minerals and Metals Limited, located in Chavara, is one of the largest industries which exports mineral sand containing mainly titanium. The soil samples were collected from this region where the industrial wastes were discharged into the lagoon. These samples were used for the screening of PAH's degrading bacteria.

In the preliminary screening, strain VITRSB1 alone grew on the plates supplemented with naphthalene, kerosene, diesel, toluene, and aniline and thus it was chosen for further studies. Nonetheless, the growth of VITRSB1 was completely inhibited when the media is supplemented with phenol, camphor, and DAP.

**Figure 1:**
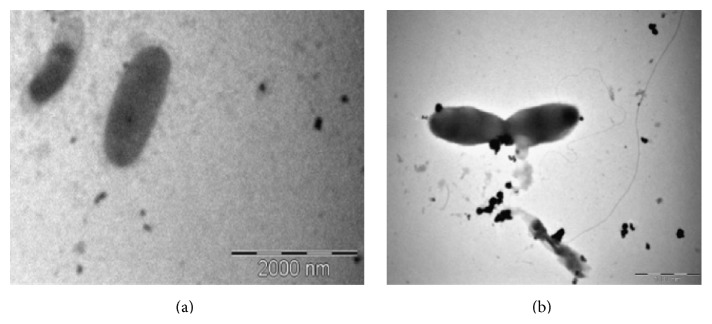
TEM image of* Burkholderia *sp. VITRSB1 with single flagellum at the end of the bacteria. Samples were stained with osmium tetroxide. Magnification: 17500x; (a) the rod shape of the* Burkholderia* is evident from the TEM image; (b) the flagellum is clearly visible at the end of the bacteria.

**Figure 2:**
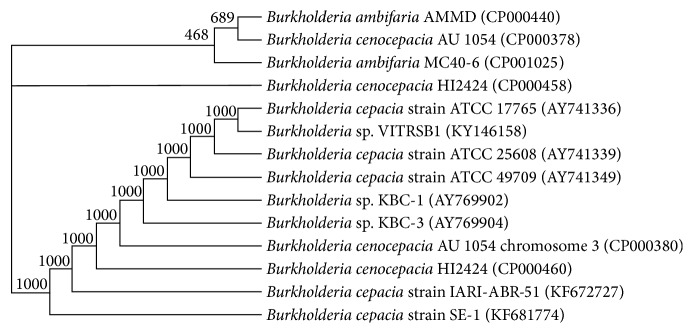
Phylogenetic tree based on 16S rRNA gene sequences displaying the position of the isolate* Burkholderia* sp. VITRSB1 with a bootstrap value of 1000. Scale bar: 0.02.

The phenotypic characters showed the strain VITRSB1 is Gram negative, aerobic, motile, and rod-shaped. The colonies were mucoid and white in colour. The size of the bacteria is about 2–5 *μ*m in length and a flagellum was observed when viewed under TEM (Figure 1). The strain utilizes sodium nitrate as nitrogen source and sodium succinate as carbon source. BLAST analysis based on the 16S rRNA gene sequence of the strain revealed 99% similarity with* Burkholderia cenocepacia* H111 strain. The phylogenetic tree of the strain showed that it occupies a distinct position within the representatives of* Burkholderia* family (Figure 2). The strain was submitted in genbank under the accession number KF146158. The G + C content was found to be 55.20%.

In the preliminary analysis, growth was observed in plates supplemented with diesel, kerosene, and toluene indicating that the PAHs were utilized by the strain. Since there was no growth in plates supplemented with higher concentration (1%) of aniline, naphthalene, and phenol, a lower concentration (0.1%) of these compounds was used for further studies. In the degradation test, the BSM containing diesel turned turbid and the colour changed to green indicating the degradation. The degradation test for toluene, aniline, kerosene, and naphthalene showed notable growth indicating the tolerance of the organism to the hydrocarbons. It has been reported that* Burkholderia* synthesize three ring fission pathways and three oxygenases for the oxidation of substituted benzene [31].* Burkholderia* also synthesize various enzymes for the degradation of aromatic hydrocarbons [32].

**Figure 3:**
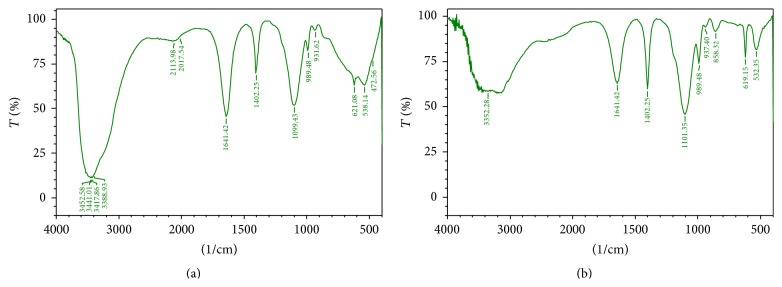
FTIR spectrum of toluene before and after degradation. About 1% toluene incubated with VITRSB1 strain for 48 hrs and analysed in FTIR. (a) Toluene before degradation with VITRSB1 strain. (b) Toluene after degradation with VITRSB1 strain.

**Figure 4:**
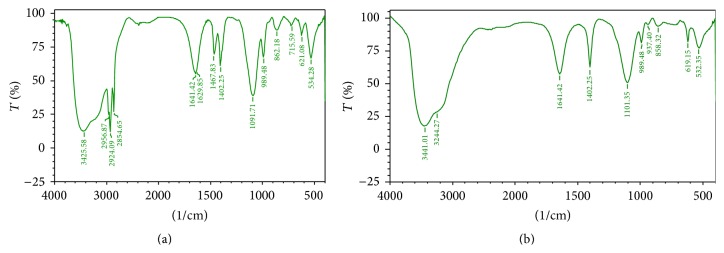
FTIR spectrum of diesel obtained before and after degradation. 1% diesel was used and incubated with the VITRSB1 strain. (a) Diesel before degradation with VITRSB1 strain. (b) Diesel after degradation with VITRSB1 strain.

**Figure 5:**
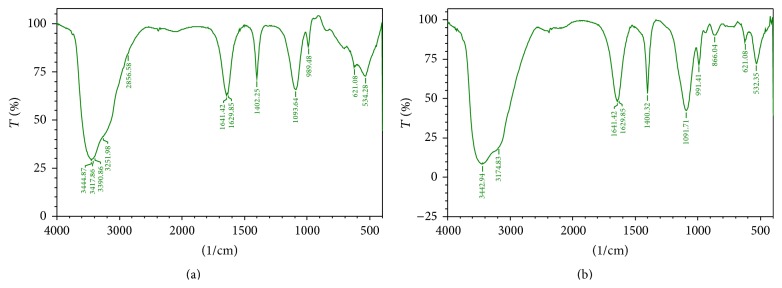
FTIR spectrum of kerosene obtained before and after degradation. 1% kerosene was used and incubated with VITRSB1 strain analysed in FTIR. (a) Kerosene before degradation with VITRSB1 strain; (b) kerosene after degradation with VITRSB1 strain.

Furthermore, the PAHs treated culture was analysed for the degraded products by FTIR. The FTIR spectra before degradation showed peaks at 3388.93, 3417.86, and 3441.01 which corresponds to the NH and OH stretch of primary amines and alcohol in the toluene (Table 1). The absence of peaks at 3388.93, 3417.86, 3441.01, 2113.98, 2017.54, 1099.43, and 621.08 after degradation indicates the degradation of toluene (Figure 3) [33]. Diesel contains hydrocarbons in the range of 2000 to 4000, a complex mixture of normal, branched, and cyclic alkanes, and aromatic compounds [34].  Figure 4 shows the degradation spectra of diesel. The peaks at 2956.87 and 2924.09 correspond to the CH, OH, and NH stretch of alkanes, alcohols, and amines (Table 2). The absence of peaks at 2956.87, 2924.09, 2854.65, 1629.85, 1467.83, and 1091.71 in the second spectrum (Figure 4) indicates the degradation of compounds. The FTIR spectra of kerosene degradation spectra (Figure 5) reveal the presence of peaks at 3251, 3444.87, 3417, and 3390 which corresponds to primary and secondary amines (Table 3). The absence of peaks in these regions after degradation indicates degradation of kerosene.

**Figure 6:**
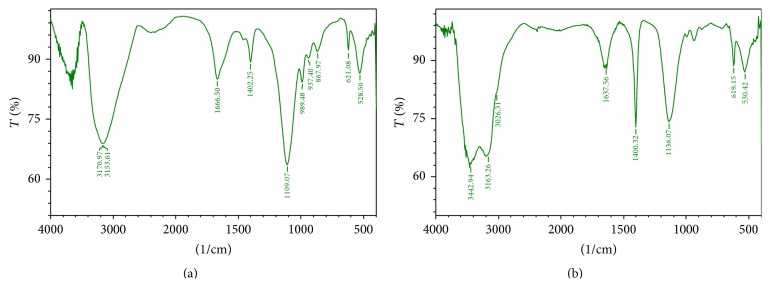
Naphthalene degradation spectra obtained before and after degradation. 1% naphthalene was used and incubated with VITRSB1 strain and analysed in FTIR. (a) Naphthalene before degradation with VITRSB1 strain; (b) naphthalene after degradation with VITRSB1 strain.

**Figure 7:**
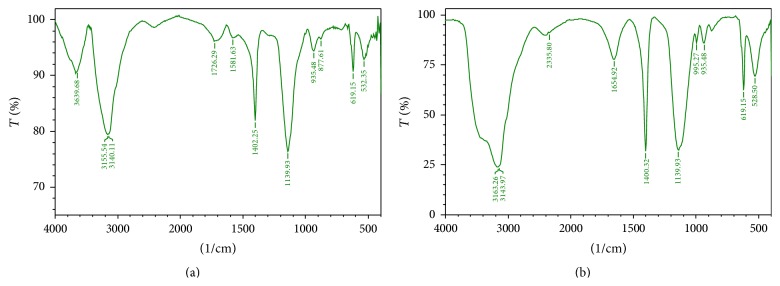
Aniline degradation spectra obtained before and after degradation. 1% aniline incubated with VITRSB1 strain and analysed in FTIR. (a) Aniline before degradation with VITRSB1 strain. (b) Aniline after degradation with VITRSB1 strain.

Naphthalene spectra (Figure 6) show peaks at 3170.97 and 1666.50 which corresponds to -C≡C-H: C-H stretch of alkynes and alkenes (Table 4) and the absence of peaks at 3170.97, 1666.50, 989.48, 937.40, 1109.07, and 867.97 after the treatment with VITRSB1 indicates the degradation of naphthalene [35]. FTIR spectra of aniline (Figure 7) show the peaks at 3639.68, 1726.29, and 1581.63 which corresponds to OH, C-O, and NH stretch of alcohols, carboxylic acids, and amines (Table 5). The absence of peaks at 3639.68, 1726.29, 1581.63, and 877.61 after treatment indicates the degradation of aniline [36, 37]. The* Burkholderia* sp. VITRSB1 did not show any growth when the BSM media were supplemented with phenol, camphor, and DAP. Growth was completely inhibited. Generally, high molecular weight PAHs degrade very slowly because the biodegradation ability primarily depends on the complexity of the chemical structures [38]. Therefore, the degradation efficiency is highly dependent on the type of hydrocarbon. This study reveals the ability of the marine derived* Burkholderia* sp. VITRSB1 to degrade low molecular weight hydrocarbons effectively. Hence, the use of* Burkholderia *will be effective and eco-friendly for the degradation of low molecular weight hydrocarbons. Further research on* in situ* bioremediation approaches and its plant growth promoting activities will reveal the potential applications of this strain.

## Figures and Tables

**Table 1:** Table showing frequencies obtained in FTIR and their corresponding functional groups before degradation and after degradation of toluene.

Serial number	Frequency	Bond	Functional group
Before degradation			

1	3388.93 3417.86 3441.01 3452.58	N H stretch O H stretch H bonded	Primary and secondary amines, amides Alcohols, phenols
2	2113.98 2017.54	C (triple bond) C stretch	Alkynes
3	1641.42	C=C stretch	Alkenes
4	1402.25	C C stretch (in ring)	Aromatics
5	1099.43	C N stretch	Aliphatic amines
6	989.48	C H bend	Alkenes
7	931.62	O H bend	Carboxylic acids
8	621.08 538.14	C Br stretch	Alkyl halides

After degradation			

1	3352.28	N H stretch O H stretch H bonded	Primary and secondary amines, amides Alcohols, phenols
2	1641.42	C=C stretch	Alkenes
3	1402.25	C C stretch (in ring)	Aromatics
4	1101.35	C O stretch	Alcohols, carboxylic acids, esters, and ethers
5	989.48	C H bend	Alkenes
6	937.40	O H bend	Carboxylic acids
7	858.32	C H “oop” N H wag =C H bend	Aromatics Primary and secondary amines Alkenes
8	532.35 619.15	C Br stretch	Alkyl halides

**Table 2:** Table showing frequencies obtained in FTIR and their corresponding functional groups before degradation and after degradation of diesel.

Serial number	Frequency	Bond	Functional group
Before degradation			

1	3425.58	O-H stretch, H bonded	Alcohol, phenols
2	2956.87 2924.09 2854.65	C-H stretch O-H stretch	Alkanes Carboxylic acids
3	1641.42	N-H bend -C=C- stretch	Primary amines Alkenes
4	1629.85	N-H bend	Primary amines
5	1467.83	C-H bend C-C stretch (in ring)	Alkanes Aromatics
6	1402.25	C-C stretch (in ring)	Aromatics
7	1091.71	C-N stretch	Aliphatic amines
8	989.48	=C-H bend	Alkenes
9	862.18	N-H wag	Primary and secondary amines
10	715.58	C-H “oop”	Aromatics
11	621.08 534.28	C-Br stretch	Alkyl halides

After degradation			

1	3441.01 3244.27	O-H stretch, H-bonded	Alcohols, phenols
2	1641.42	-C=C- stretch	Alkenes
3	1402.25	C-C stretch (in ring)	Aromatics
4	1101.35	C-H stretch	Aliphatic amines
5	989.48 937.40	=C-H bend	Alkenes
6	858.32	N-H wag	Primary and secondary amines
7	619.15 532.35	C-Br stretch	Alkyl halides

**Table 3:** Table showing frequencies visible in the spectra and their possible functional groups in FTIR before and after degradation of kerosene.

Serial number	Frequency	Bond	Functional group
Before degradation			

1	3444.87 3417.86 3390.86	N H stretch O-H stretch, H bonded	Primary and secondary amines, amides Alcohols, phenols
2	3251.98	N H stretch C≡C H:CH stretch	Primary and secondary amines, amides
3	2856.58	C H stretch O H stretch	Alkanes Carboxylic acids
4	1641.42	C=C stretch N H bend	Alkenes Primary amines
5	1629.85	N H bend C=C stretch	Primary amines Alkenes
6	1402.25	C C stretch (in ring)	Aromatics
7	1093.64	C N stretch	Aliphatic amines
8	989.48	=C H bend	Alkenes
9	621.08	C Cl stretch C≡C H:C H B end	Alkyl halides Alkynes
10	534.28	C Br stretch	Alkyl halides

After degradation			

1	3442.94	O H stretch, H bonded	Alcohols, phenols
2	3174.83	O H stretch	Carboxylic acids
3	1641.42	C=C stretch N H bend	Alkenes Primary amines
4	1629.85	N H bend	Primary amines
5	1400.32	C C stretch (in ring)	Aromatics
6	1091.71	C O stretch	Alcohols, carboxylic acids, esters, and ethers
7	991.41	=C H bend	Alkenes
8	866.04	N H wag	Primary and secondary amines
9	621.08	C Cl stretch	Alkyl halides
10	532.35	C Br stretch	Alkyl halides

**Table 4:** Table showing frequencies visible in the spectra and their possible functional groups before and after degradation of naphthalene.

Serial number	Frequency	Bond	Functional group
Before degradation			

1	3170.97 3153.61	-C≡C-H:C-H stretch	Alkynes (terminal)
2	1666.50	-C=C- stretch	Alkenes
3	1402.25	C-C stretch (in ring)	Aromatics
4	1109.07	C-N stretch	Aliphatic amines
5	989.48 937.40	=C-H bend	Alkenes
6	867.97	C-H “oop”	Aromatics
7	621.08 528.50	C-Br stretch	Alkyl halides

After degradation			

1	3442.94	O-H stretch, H bonded	Alcohols, phenols
2	3163.26	O-H stretch	Carboxylic acids
3	3026.31	C-H stretch	Aromatics
4	1637.56	N-H bend	Primary amines
5	1400.32	C-C stretch (in ring)	Aromatics
6	1136.07	C-H rock	Alkanes
7	616.15 530.42	C-Br stretch	Alkyl halides

**Table 5:** Table showing frequencies visible in the spectra and their possible functional groups before and after degradation of aniline.

Serial number	Frequency	Bond	Functional group
Before degradation			

1	3639.68	O-H stretch, free hydroxyl	Alcohols, phenols
2	3155.54 3140.11	O-H stretch	Carboxylic acids
3	1726.29	C=O stretch	Carbonyls, carboxylic acids, aldehydes, saturated aliphatics, and *α* and *β*-unsaturated esters
4	1581.63	N-H bend	Primary amines
5	1402.25	C-C stretch (in ring)	Aromatics
6	1139.93	C-O stretch	Alcohols, carboxylic acids, esters, and ethers
7	935.48	=C-H bend	Alkenes
8	877.61	C-H “oop”	Aromatics
9	619.15 532.35	C-Br stretch	Alkyl halides

After degradation			

1	3163.26 3143.97	O-H stretch	Carboxylic acids
2	1654.92	-C=C- stretch	Alkenes
3	1400.32	C-C stretch (in ring)	Aromatics
4	1139.93	C-O stretch	Alcohols, carboxylic acids, esters, and ethers
5	995.27 935.48	=C-H bend	Alkenes
6	619.15 528.50	C-Br stretch	Alkyl halides
