# Corrigendum to “Biodegradation of PAHs by *Burkholderia* sp. VITRSB1 Isolated from Marine Sediments”

**DOI:** 10.1155/2019/1513982

**Published:** 2019-12-04

**Authors:** T. Revathy, M. A. Jayasri, K. Suthindhiran

**Affiliations:** Marine Biotechnology and Bioproducts Laboratory, School of Biosciences and Technology, VIT University, Vellore, Tamil Nadu 632014, India

In the article titled “Biodegradation of PAHs by *Burkholderia* sp. VITRSB1 Isolated from Marine Sediments” [1], there is an error in the Results and Discussion section where the text reading “PAHs are group of compounds composed of fused aromatic rings that are highly stable and carcinogenic” should be corrected to “Some PAH compounds and their metabolites are highly stable and carcinogenic.”
